# The Relationship Between Physical Activity Profiles and Cardiovascular Disease Risk Factors: Results of a Cross-Sectional Survey of Active Duty U.S. Service Members

**DOI:** 10.1093/milmed/usae381

**Published:** 2024-08-02

**Authors:** Jimmy Dawood, James D Mancuso, Kasi Chu, Martin Ottolini, Anwar E Ahmed

**Affiliations:** F. Edward Hebert School of Medicine, Uniformed Services University of the Health Sciences, Bethesda, MD 20814, USA; Department of General Surgery, Naval Medical Center Portsmouth, Portsmouth, VA 23708, USA; Department of Preventive Medicine & Biostatistics, Uniformed Services University of the Health Sciences, Bethesda, MD 20814, USA; Department of Preventive Medicine & Biostatistics, Uniformed Services University of the Health Sciences, Bethesda, MD 20814, USA; F. Edward Hebert School of Medicine, Uniformed Services University of the Health Sciences, Bethesda, MD 20814, USA; Department of Preventive Medicine & Biostatistics, Uniformed Services University of the Health Sciences, Bethesda, MD 20814, USA

## Abstract

**Introduction:**

This study aimed to identify subgroups of active duty U.S. service members (ADSMs) based on physical activity levels and their association with cardiovascular disease (CVD) risk factors. Our secondary aim was to assess how these profiles vary across sociodemographic factors.

**Methods:**

A cross-sectional survey of ADSMs, yielding a 9.6% response rate and 17,166 usable surveys, was conducted by the DoD and RAND Corporation in 2018 using stratified random sampling. In this secondary analysis, latent subgroups of ADSMs were determined based on physical activity levels and a weighted multinomial logistic regression was used to examine associations.

**Results:**

Three latent subgroups were identified as “High Activity” (17.1%), “Moderate Activity” (45.3%), and “Low Active” (37.6%). Older age, female, White (as compared to Hispanic), cohabiting, Air Force, Navy, and Coast Guard were associated with increased odds of “Low Active” membership. Compared to the “Low Active” class, the “High Active” class showed lower odds of hyperlipidemia (aOR = 0.62, 95% CI: 0.38, 0.99), hypertension (aOR = 0.69, 95% CI: 0.48, 0.98), and multimorbidity (aOR = 0.55, 95% CI: 0.38, 0.80). Compared to the “Low Active” class, the “Moderate Active” class showed lower odds of hyperlipidemia (aOR = 0.62, 95% CI: 0.47, 0.81) and multimorbidity (aOR = 0.66, 95% CI: 0.53, 0.83). Similar patterns of associations were seen in ADSMs who met the objectives for Healthy People 2030 (HP2030) standards.

**Conclusions:**

The study emphasizes the importance of combining physical activity and strength training to reduce CVD risk factors, supporting the implementation of tailored physical activity programs within the military to align fitness standards.

## INTRODUCTION

Hypertension, hyperlipidemia, and obesity are the risk factors that are positively associated with increasing prevalence of cardiovascular disease (CVD) and serve as quantitative metrics in monitoring its progression.^1^ CVD remains the leading cause of morbidity and mortality in the United States, contributing to the death of approximately 610,000 people per year.^2^ According to the American College of Cardiology (ACC) and the American Heart Association (AHA) 2017 guidelines, the absolute burden of hypertension has significantly increased in the last two decades in the U.S. general population.^3^ Hypertension alone was also a primary or contributing cause to the premature death of over 500,000 Americans in 2019.^4^ Obesity and hyperlipidemia are both associated and independent risk factors for hypertension, with all three comorbidities commonly found in populations at risk for cardiovascular complications.^2,5^

The Office of Disease Prevention and Health Promotion (ODPHP) releases a set of data driven national objectives every decade, the most recent entitled Healthy People 2030 (HP2030). The objectives contain recommendations for aerobic and muscle-strengthening activity for substantial and extensive health benefits. These recommendations are not only applicable to the civilian population, but also to active duty U.S. service members (ADSM) as well. The U.S. Military has branch-specific standards for physical fitness and height and weight that are enforced with periodic physical fitness and health assessments. Meanwhile ADSMs across all branches far exceeded the goals outlined by HP2030 for both aerobic and strength-training recommendations based on 2018 data.^6^ However, the rising prevalence of obesity among the current ADSM population as well as the decreasing availability of potential recruits from a healthy selection pool remain an ongoing issue and has the potential to limit the DoD’s ability to sustain a fully capable and ready military force.^7^ The association between physical activity and the development of certain CVD risk factors that include hypertension, hyperlipidemia, and obesity is well established in the civilian population. However, these associations have not been fully explored in the U.S. ADSM population in relation to HP2030 physical activity goals. Analyses in this investigation were based on secondary data from the Health-Related Behavior Survey (HRBS) of ADSMs, a cross-sectional survey conducted by the DoD and RAND CorporationSSS in 2018.

The ADSM population is comprised of a diverse population pool that spans multiple ages, self-identified race/ethnicities, sex, socioeconomic status, and levels of education. These sociodemographic factors exert a substantial influence on CVD risk and shape patterns of risk factor prevalence, health care utilization, and disparities within the population.^8^ Addressing these sociodemographic determinants is crucial for implementing effective preventive strategies and reducing the burden of CVD risk across diverse communities. Thus, the primary aim of this study was to identify profiles of ADSMs’ physical activity level and examine their association with specific CVD risk factors. Meanwhile, the secondary aim was to assess how these profiles varied across sociodemographic factors.

## METHODS

### Study Population

This was a representative cross-sectional survey of active component service members of the U.S. Army, Marine Corps, Navy, Air Force, and Coast Guard. It was based on a random sampling of ADSMs stratified by service branch, pay grade, and sex. The study analyzed secondary data from the 2018 HRBS. The survey was a cross-sectional instrument, developed by the DoD in 1980, that collects data triennially on the health and health-related behaviors of DoD personnel. The overall weighted response rate for the survey was 9.6%, yielding a total of 17,166 usable surveys that comprised the final analytical sample.^9^ The data incorporated post-stratification analytical weights, which accounted for the design effect (e.g., unequal probabilities of selection) and a nonresponse bias. The study was approved by the Uniformed Services University Institutional Review Board: Protocol DBS.2020.074.

### Measures

#### Physical activity

Aerobic and muscle-strengthening activities were defined as the duration (<20, 20–29, 30–59, and 60 min or more per day) and frequency (not at all, <1, 1–2, 3–4, 5–6, and about every day per week) of physical activity over the previous 30 days.^9^ We used these definitions: moderate physical activity (MPA) elevates the heart rate and breathing but allows the individual to maintain a conversation; vigorous physical activity (VPA) is exertion which does not allow participants to maintain a conversation; and strength training is using weights or resistance to increase muscle strength. Latent subgroups were determined based on duration (60 min or more per day: “yes/no”) and frequency (3 days or more per week “yes/no”) of moderate and vigorous aerobic and muscle-strengthening activities. These classifications have been described in the existing HRBS literature.^9^ We further categorized physical activity according to the objectives defined by the ODPHP in their HP2030 objectives:

(1) Substantial health benefits from moderate or vigorous physical activity (MVPA) defined as MPA at least 150 min per week or VPA at least 75 min per week (yes/no).^9^

(2) Extensive health benefits from MVPA defined as MPA at least 300 min per week or VPA at least 150 min per week (yes/no).^9^

#### Study outcome

Participants were categorized as having hypertension (yes/no) or hyperlipidemia (yes/no) if they had been diagnosed by a doctor or health professional in the past 12 months. Obesity was defined as a current body mass index of 30.0 kg/m^2^ or more (yes/no).^9^ The study outcomes consisted of five distinct health condition outcome categories: (1) none “reference,” (2) hyperlipidemia only, (3) hypertension only, (4) obesity only, and (5) presence of two or more of the aforementioned CVD risk factors (multimorbidity).

#### Demographic factors

Study socio-demographic factors included sex (male, female), self-identified race/ethnicity (White, Black, Hispanic, Other), age group (17–24, 25–34, 35+), education (high school or less, some college, bachelor’s degree or more), marital status (married, cohabiting, never married, separated/divorced/widowed), service branch (Air Force, Army, Marine Corps, Navy, Coast Guard), and rank (enlisted, officer).

#### Statistical analysis

Data analysis was conducted using SAS 9.4 (SAS Institute Inc., Cary, NC, USA). Survey weights were applied in all calculations to generate estimates representative of the military’s active duty population. Ninety-five percent confidence intervals (CIs) and unadjusted associations were tested using Rao-Scott chi-squared tests. Latent cluster analysis (LCA), a person-centered approach, was employed to identify subgroups of ADSMs by modeling their patterns of physical activity (duration/day and frequency/week) during the past 30 days. A suitable number of subpopulations was determined by the average class assignment probabilities. A solution with high diagonal probabilities of greater than 0.80 indicates the best-fit model. Differences in sociodemographic factors were examined across the physical activity profiles by a weighted multinomial logistic regression model. The associations between physical activity profiles and CVD risk factors were evaluated by a weighted multinomial logistic regression model, which controlled for sociodemographic factors (age, gender, race, education, pay grade, service branch, and marital status). We also evaluated the relationship between substantial MVPA, extensive MVPA, and single physical activity components and CVD risk factors. All associations were assessed at a significance level of 0.05.

## RESULTS

**Figure 1.**
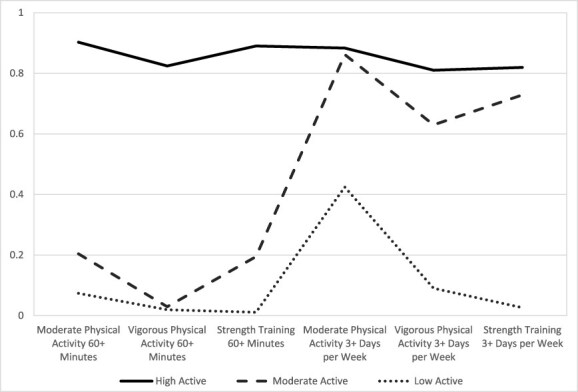
The conditional probabilities of physical activity items across different classes.

Overall, the 2018 HRBS-weighted prevalence estimates of hypertension, hyperlipidemia, and obesity were 9.1% (95% CI: 8.45–9.78%), 4.2% (95% CI: 3.81–4.55%), and 14.4% (95% CI: 13.54–15.17%) respectively (**Table 1**). The prevalence of multimorbidity was 4.5% (95% CI: 4.06–4.92%). Among respondents, 71.1%, 47.0%, and 49.6% of service members reported engaging in 3 or more days a week of MPA, VPA, and strength training, respectively. HP2030 physical activity objectives for substantial benefits from MVPA were met by 71.8% and extensive benefits from MVPA by 45.3%. Latent class analysis identified three subpopulations of ADSMs, described as high level of physical activity or “High Active” (17.1%), moderate level of physical activity or “Moderate Active” (45.3%), and low level of physical activity or “Low Active” (37.6%). ADSMs belonging to the “High Active” class showed the highest conditional probability to all six physical activity items (Figure 1). Air Force (“Low Active,” 4.6%; “Moderate Active,” 2.5%; “High Active,” 2.1%, *P* = .0004) and Navy (“Low Active,” 6.3%; “Moderate Active,” 5.2%; “High Active,” 3.5%; *P* = .0074) service members belonging to the “Low Active” class have significantly higher prevalence of multimorbidity than service members belonging to the “High Active” class.

**Table 1.** Descriptive statistics of chronic conditions, sociodemographic factors, and physical activity in ADSMs, 2018 (*N* = 17,166).

	Levels		95% Confidence Limits	
		*n* (%)	Lower	Upper
Hypertension	Yes	1,749 (9.1)	8.5	9.8
Hyperlipidemia	Yes	1,076 (4.2)	3.8	4.6
Obesity	Yes	2,467 (14.4)	13.5	15.2
Sex	Female	5,353 (16.7)	16.0	17.4
	Male	11,813 (83.3)	82.6	84.0
Age (years)	17–24	3,642 (37.8)	36.4	39.1
	25–34	6,467 (39.9)	38.8	41.1
	35+	7,057 (22.3)	21.6	23.0
Education	Missing	250 (1.2)	0.9	1.4
	High school or less	7,990 (64.4)	63.4	65.4
	Some college	2,625 (12.8)	12.2	13.5
	Bachelor’s degree or more	6,301 (21.6)	20.9	22.4
Race	Missing	68 (0.7)	0.4	1.0
	Black	2,226 (16.2)	15.2	17.1
	Hispanic	2,459 (16.0)	15.1	16.9
	Other	1,747 (9.5)	8.8	10.1
	White	10,666 (57.6)	56.4	58.9
Marital status	Married	10,776 (53.8)	52.6	55.1
	Cohabiting	1,042 (7.8)	7.1	8.5
	Separated, divorced, or widowed	1,284 (6.3)	5.7	6.8
	Never married	4,064 (32.1)	30.9	33.4
Service branch	Air Force	5,579 (24.1)	23.3	24.9
	Army	3,646 (34.5)	33.2	35.8
	Marine Corps	2,569 (13.9)	13.1	14.7
	Navy	3,675 (24.4)	23.3	25.4
	Coast Guard	1,697 (3.2)	3.0	3.4
Pay grade	Enlisted	12,154 (83.5)	82.9	84.1
	Officer	5,012 (16.5)	15.9	17.1
MPA 60+ min	No	13,079 (72.0)	70.9	73.2
	Yes	4,087 (28.0)	26.8	29.1
VPA 60+ min	No	15,016 (83.6)	82.6	84.6
	Yes	2,150 (16.4)	15.4	17.4
Strength training 60+ min	No	13,905 (74.9)	73.8	76.1
	Yes	3,261 (25.1)	23.9	26.2
MPA 3+ days per week	No	5,423 (28.9)	27.8	30.0
	Yes	11,743 (71.1)	70.0	72.2
VPA 3+ days per week	No	9,604 (53.0)	51.7	54.2
	Yes	7,562 (47.0)	45.8	48.3
Strength training 3+ days per week	No	9,541 (50.4)	49.2	51.6
	Yes	7,625 (49.6)	48.4	50.8
Physical activity profiles	Low active	7,367 (37.6)	36.4	38.7
	Moderate active	7,626 (45.3)	44.1	46.5
	High active	2,173 (17.1)	16.1	18.2
HP2030 goal				
Substantial MVPA (MPA at least 150 min or VPA at least 75 min per week)	No	5,264 (28.2)	27.1	29.2
	Yes	11,902 (71.8)	70.8	72.9
Extensive MVPA (MPA at least 300 min or VPA at least 150 min per week)	No	10,141 (54.7)	53.4	55.9
	Yes	7,025 (45.3)	44.1	46.6

MVPA = Moderate of Vigorous Physical Activity.

MPA = Moderate Physical Activity.

VPA = Vigorous Physical Activity.

ADSM = Active-Duty Service Member.

HP2030 = Healthy People 2030.

Latent class analysis identified three subpopulations of active-duty service members (ADSMs), described as High Active’ or high level of physical activity (17.1%), “Moderate Active” or moderate level of physical activity (45.3%), and “Low Active” or low level of physical activity (37.6%). ADSMs belonging to “high level of physical activity’ class showed the highest conditional probability to all six physical activity items. ADSMs belonging to “low level of physical activity” class showed the lowest conditional probability to all six physical activity items.

In bivariate analysis, we found significant disparities in CVD risk factors across sociodemographic and military characteristics. The prevalence estimates of CVD risk factors varied by sex, age groups, education level, race, marital status, service branch, and pay grade. The prevalence estimates of CVD risk factors decreased among respondents who engaged in physical activity for 3 or more days in a week and for 60 or more minutes a day of MPA, VPA, and strength training during the past 30 days. Significant prevalence differences were found in ADSMs belonging to the “High Active” class and “Low Active” class.

**Table 2** illustrates the estimated 2018 HRBS adjusted odds ratios (aOR) in the multinomial logistic regression models analyzing the associations between physical activity profiles (with “Moderate Active” being the reference). Older age, female, White (as compared to Hispanic), cohabiting, the Air Force, the Navy, and the Coast Guard were associated with increased odds of “Low Active” membership. Older age, female, the Air Force, the Marine Corps, the Navy, and the Coast Guard were associated with decreased odds of “High Active” membership. For instance, being an Air Force service member versus an Army service member increases the odds of being in “Low Active” class versus “Moderate Active” class by a factor of 1.42 (aOR = 1.42, 95% CI: 1.25–1.62) and decreases the odds of being in “High Active” class versus “Moderate Active” class by a factor of 0.68 (aOR = 0.68, 95% CI: 0.57–0.82).

**Table 2.** Adjusted odds ratio of physical activity profiles (ref = moderate active) by sociodemographic factors among ADSMs, 2018.

		High Active			Low Active		
		OR	LCL	UCL	OR	LCL	UCL
Age (years)	17–24	1.00			1.00		
	25–34	**0.70**	**0.56**	**0.86**	0.95	0.82	1.12
	35+	**0.54**	**0.42**	**0.68**	**1.20**	**1.01**	**1.43**
Sex	Female	**0.60**	**0.50**	**0.72**	**1.71**	**1.53**	**1.91**
	Male	1.00			1.00		
Race	Black	1.13	0.90	1.42	0.89	0.76	1.06
	Hispanic	1.00	0.80	1.26	**0.78**	**0.67**	**0.91**
	Other	1.22	0.94	1.57	0.95	0.80	1.12
	White	1.00			1.00		
Pay grade	Enlisted	1.00			1.00		
	Officer	0.81	0.65	1.01	1.05	0.92	1.21
Education	High school or less	1.00			1.00		
	Some college	**0.80**	**0.65**	**0.99**	**0.83**	**0.72**	**0.95**
	Bachelor’s degree or more	0.89	0.71	1.11	0.88	0.76	1.02
Service branch	Air Force	**0.68**	**0.57**	**0.82**	**1.42**	**1.25**	**1.62**
	Army	1.00			1.00		
	Marine Corps	**0.65**	**0.52**	**0.83**	0.98	0.81	1.18
	Navy	**0.74**	**0.57**	**0.94**	**2.00**	**1.70**	**2.36**
	Coast Guard	**0.74**	**0.58**	**0.94**	**1.67**	**1.41**	**1.99**
Marital status	Married	0.99	0.71	1.37	1.14	0.90	1.44
	Cohabiting	0.98	0.80	1.21	**1.25**	**1.08**	**1.44**
	Separated, divorced, or widowed	**1.43**	**1.04**	**1.99**	1.02	0.82	1.26
	Never Married	1.00			1.00		

Boldface indicates statistical significance (*P*=.05).

**Table 3** illustrates the estimated 2018 HRBS aOR in the multinomial logistic regression models analyzing the associations between physical activity profiles and CVD risk factors, adjusting for sex, race, age groups, education, marital status, service branch, and pay grade. Participating in moderate physical activity 3 days or more a week was associated with lower odds of multimorbidity (aOR = 0.65, 95% CI: 0.52–0.81). Participating in vigorous physical activity 3 or more days a week was associated with significantly lower odds of hyperlipidemia (aOR = 0.73, 95% CI: 0.57–0.95) and multimorbidity (aOR = 0.72, 95% CI: 0.57–0.89). Strength training of 60 min or more per day was associated with significantly lower odds of hypertension (aOR = 0.69, 95% CI: 0.52–0.93) and multimorbidity (aOR = 0.71, 95% CI: 0.52–0.97). Strength training 3 days or more per week was associated with significantly lower odds of hyperlipidemia (aOR = 0.68, 95% CI: 0.52–0.89), hypertension (aOR = 0.70, 95% CI: 0.56–0.88), and multimorbidity (aOR = 0.74, 95% CI: 0.60–0.93).

**Table 3.** Adjusted odds ratio of chronic conditions by physical activity among ADSMs, controlling for sociodemographic factors, 2018.

		Hyperlipidemia only			Hypertension only			Obese only			Multimorbidity		
	Levels	OR	LCL	UCL	OR	LCL	UCL	OR	LCL	UCL	OR	LCL	UCL
MPA 60+ min	Yes	0.97	0.70	1.34	0.95	0.73	1.23	0.86	0.71	1.04	0.80	0.62	1.03
	No	1.00			1.00			1.00			1.00		
VPA 60+ min	Yes	0.76	0.49	1.20	0.80	0.57	1.13	0.94	0.74	1.20	0.72	0.50	1.04
	No	1.00			1.00			1.00			1.00		
Strength training 60+ min	Yes	0.69	0.46	1.04	**0.69**	**0.52**	**0.93**	1.02	0.83	1.24	**0.71**	**0.52**	**0.97**
	No	1.00			1.00			1.00			1.00		
MPA 3+ days per week	Yes	0.82	0.63	1.06	0.86	0.67	1.10	0.90	0.76	1.07	**0.65**	**0.52**	**0.81**
	No	1.00			1.00			1.00			1.00		
VPA 3+ days per week	Yes	**0.73**	**0.57**	**0.95**	0.84	0.66	1.05	0.96	0.82	1.12	**0.72**	**0.57**	**0.89**
	No	1.00			1.00			1.00			1.00		
Strength training 3+ days per week	Yes	**0.68**	**0.52**	**0.89**	**0.70**	**0.56**	**0.88**	1.01	0.86	1.18	**0.74**	**0.60**	**0.93**
	No	1.00			1.00			1.00			1.00		
Physical activity profiles	Low Active	1.00			1.00			1.00			1.00		
	Moderate Active	**0.62**	**0.47**	**0.81**	0.80	0.63	1.02	0.96	0.81	1.14	**0.66**	**0.53**	**0.83**
	High Active	**0.62**	**0.38**	**0.99**	**0.69**	**0.48**	**0.98**	0.96	0.74	1.24	**0.55**	**0.38**	**0.80**
HP2030 goal													
Substantial MVPA (MPA at least 150 min or VPA at least 75 min per week)	No	1.00			1.00			1.00			1.00		
	Yes	**0.67**	**0.50**	**0.88**	0.77	0.59	1.01	0.90	0.76	1.08	**0.55**	**0.45**	**0.69**
Extensive MVPA (MPA at least 300 min or VPA at least 150 min per week)	No	1.00			1.00			1.00			1.00		
	Yes	**0.73**	**0.56**	**0.95**	**0.75**	**0.60**	**0.94**	0.87	0.74	1.02	**0.64**	**0.51**	**0.80**
Combined effects of substantial MVPA and 3+ days of strength training per week													
	Neither	1.00			1.00			1.00			1.00		
	Strength training only	**0.35**	**0.17**	**0.73**	0.50	0.25	1.01	0.94	0.62	1.42	0.95	0.57	1.56
	Substantial MVPA only	**0.64**	**0.45**	**0.89**	0.79	0.57	1.09	0.85	0.68	1.06	**0.59**	**0.45**	**0.76**
	Both	**0.55**	**0.40**	**0.76**	**0.62**	**0.47**	**0.84**	0.92	0.75	1.13	**0.52**	**0.40**	**0.68**

Each covariate was evaluated by a weighted multinomial logistic regression model, controlling for sociodemographic factors (age, gender, race, education, pay grade, service branch, and marital status). MVPA = Moderate of Vigorous Physical Activity; MPA = Moderate Physical Activity; VPA = Vigorous Physical Activity; ADSM = Active-Duty Service Member; HP2030 = Healthy People 2030.

Boldface indicates statistical significance (*P*=.05).

Compared to the “Low Active” class, the “High Active” showed lower odds of hyperlipidemia (aOR = 0.62, 95% CI: 0.38–0.99), hypertension (aOR = 0.69, 95% CI: 0.48–0.98), and multimorbidity (aOR = 0.55, 95% CI: 0.38–0.80). Compared to the “Low Active” class, the “Moderate Active” class showed lower odds of hyperlipidemia (aOR = 0.62, 95% CI: 0.47–0.81) and multimorbidity (aOR = 0.66, 95% CI: 0.53–0.83). Similar patterns of associations were seen in ADSMs who met the HP2030 standards. ADSMs who met the HP2030 standards for at least 150 min per week of MPA or at least 75 min per week of VPA had significantly lower odds of hyperlipidemia (aOR = 0.67, 95% CI: 0.50–0.88) and multimorbidity (aOR = 0.55, 95% CI: 0.45–0.69). ADSMs who met the 300 min/week of MPA or at least 150 min/week of VPA had significantly lower odds of hyperlipidemia (aOR = 0.73, 95% CI: 0.56–0.95), hypertension (aOR = 0.75, 95% CI: 0.60–0.94), and multimorbidity (aOR = 0.64, 95% CI: 0.51–0.80). In the combined model that included both substantial MVPA and 3+ days of strength training per week, those who did both had significantly lower prevalence of hyperlipidemia, hypertension, and multimorbidity. In contrast, those who had substantial MVPA only had lower prevalence of hyperlipidemia and multimorbidity only; those who did strength training only had a lower prevalence of hyperlipidemia but not the other outcomes.

## DISCUSSION

In this study, we provide valuable insight into the association between physical activity profiles of ADSMs and the prevalence of hypertension, hyperlipidemia, obesity, and multimorbidity. The combination of substantial physical activity and strength training was associated with a lower prevalence of CVD risk factors compared to either alone. These findings are vital in understanding the health status of the military population and may have implications for military readiness and health care planning. Notably, the substantial prevalence of hypertension, a leading contributor to cardiovascular morbidity and mortality, underscores the gravity of cardiovascular health concerns within the military.^2^ This aligns with broader societal trends of increasing prevalence of hypertension and prompts a closer examination of the distinctive factors influencing cardiovascular health of ADSMs.^10^

The application of LCA to discern three distinct physical activity profiles among ADSMs provides a nuanced perspective on the diverse exercise habits within the U.S. military. The “High Active” subgroup (17.1%) demonstrated the highest conditional probability for engaging in all six assessed physical activity components and demonstrated significantly lower prevalence of all CVD risk factors. This finding supports the potential protective effects of a comprehensive and sustained high-level physical activity regimen. The “Moderate Active” subgroup (45.3%) also demonstrated favorable association with lower prevalence estimates of CVD risk factors. This finding supports the protective and often underestimated health impact of a modest amount of physical activity performed at an adequate intensity, duration, and frequency.^11^ The “Low Active” subgroup (37.6%) was associated with a higher prevalence of CVD risk factors and may warrant a closer investigation into the barriers and lifestyle factors that may be contributing to decreased activity levels.

As expected, the prevalence of physical activity levels meeting the HP2030 goals for substantial health benefits was greater among ADSM compared to the general U.S. population (71.8% vs. 26% and 19% for men and women, respectively), as was the prevalence of activity meeting aerobic activity goals for extensive health benefits (45.3% vs. 29.9%).^12^ The association between sociodemographic factors and physical activity profiles explored in this study provides contextual insight into external factors that are shaping exercise habits among ADSMs. The association between older age, female, and increased odds of belonging to the “Low Active” subgroup underscores the importance of tailoring fitness programs to address the unique challenges faced by different age groups and genders. These associations were also observed in the civilian population. Survey data from 2018 National Health and Nutrition Examination Survey (NHANES) data revealed that females had 31% lower odds of participating in sufficient amount of physical activity, meanwhile U.S. adults aged 45 to 64 years had 28% lower odds than their 18-44 year-old counterparts.^13^ The increased likelihood of White ADSMs (compared to Hispanics), cohabitants, and those serving in the Air Force, Navy, and Coast Guard belonging to the “Low Active” class also unveils potential factors associated with in physical activity engagement. Variations in physical activity in these demographics make them more susceptible to the development of the measured CVD risk factors. Tailoring interventions to reduce racial, age, and gender barriers, and service specific branch dynamics is crucial for achieving equitable health outcomes across all branches and demographics of the U.S. Military.

Multinomial logistic regression models were used to aid in understanding the relationship between different classifications of physical activity and the prevalence of CVD risk factors among ADSMs. The inverse association between engaging in MPA 3 or more days per week and lower odds of multimorbidity aligns with established ODPHP recommendations for the incorporation of moderate-intensity aerobic activity for holistic health benefits. The significant correlation between the engagement in VPA 3 or more days per week and lower odds of hyperlipidemia and multimorbidity also support its utility against CVD and multimorbidity. Meanwhile strength training consistently shows an association without specifying duration and intensity with lower odds of hypertension and multimorbidity, emphasizing the pivotal role of resistance training in promoting cardiovascular health among ADSMs. Although these associations were analyzed independently, combination training is generally considered to have a synergistic effect on health through improvement of both cardiorespiratory fitness and muscle strength.^14^ Thereby providing multimodal protective benefits for CVD and pathology as a consequence of a weakened musculoskeletal system to include weakness, falls, osteopenia, and osteoporosis. These considerations are particularly important when recommending training regimens to individuals that may have a job-specific need for these benefits or that are more susceptible to the development of the later sequela of inadequate aerobic exercise or strength training such as older individuals or women.

The observed associations between the prevalent CVD risk factors and physical activity profiles, different categories of physical activity, and sociodemographic factors provide an opportunity for tailored interventions that can optimize the health and operational effectiveness of the U.S. Military. Understanding the unique needs and obstacles of different subgroups, such as older individuals, female, specific racial and service branches, is pivotal for designing tailored interventions. Addressing these diverse demographics with targeted fitness programs and health education initiatives could contribute to more equitable health outcomes. The study results highlighting the unique associations between different categories of physical activity and CVD risk factors suggests that diverse fitness plans can be developed and tailored based on risk factors for certain CVD risk factors while still optimizing their health holistically.

## LIMITATIONS

Major limitations of this study include the low response rate and accompanying possibility of selection bias. Findings were based on a cross-sectional design making the temporality of these associations uncertain, and the self-reported survey responses introduce the possibility of misclassification bias due social desirability of certain responses (such as lower weight and greater levels of physical activity) and other factors. The active duty military personnel who took part in the HRBS contributed 41.2% of usable surveys and were predominantly comprised of officers. This is a notable contrast to the 17.7% figure seen across the DoD.^15^ Discrepancies in representation become more evident when examining individual branches, with the USMC displaying a mere 11.5% officer composition. The USMC also had a significantly higher percentage of survey participants within the age range of 17 to 24 years old (60.6%) compared to the other branches and the smallest percentage of participants over 35 years of age (11.9%).^9^ These discrepancies may suggest that the data collected by the usable surveys are not entirely generalizable to the DoD population and may have attributed to unexpected findings such as the USMC having low odds of being a part of the “High Activity” class compared to the Army. Comparison of the prevalence estimates from 2018 with earlier surveys are limited by the higher response rates in prior years as well as the differences in the survey questions. For example, the questions we used from the 2018 HRBS on chronic diseases such as hypertension were limited to a self-reported diagnosis “in the past 12 months,” whereas information from prior surveys was based on whether they had been diagnosed within the past 2 years. Direct comparisons of physical activity prevalence between ADSM and the general population were confounded by age, sex, and other demographics; as well as selection bias from the healthy soldier effect.^16^ Additionally, the assessment of chronic disease outcomes of hypertension and hyperlipidemia by self-reported diagnosis would be expected to underdiagnose the true prevalence of these outcomes compared to studies that assessed these outcomes laboratory or clinical measurement, such as NHANES. These differing methods may have resulted in different associations between these outcomes and physical activity and other exposures. The quantitative metric of obesity was determined based on BMI, a measurement that involves self-reported weight and height which may be misreported. Additionally, BMI may be an inaccurate indication of obesity in participants derived from an active duty military population that have a disproportional muscle to fat ratio compared to the civilian population. Finally, the findings from this study may not be generalizable to the general population due to differences in demographics (e.g., age, sex), as well as physical and medical requirements for military service.

## CONCLUSION

This study’s comprehensive examination of physical activity profiles and categories, and sociodemographic factors among ADSMs sheds light on various elements associated with the observed CVD risk factors. Understanding the subtleties outlined by this research can aid in the development of guided targeted interventions, refining military fitness standards, and promoting a resilient and healthy active duty force. Actionable goals may include further research aimed at identifying the barriers or limitations that are being met by ADSMs that are identified with sociodemographic factors associated with decreased activity and thereby a higher prevalence of specific CVD risk factors. Once those limitations are identified, strategies can be developed and employed aimed at targeting them. Similar strategies that focus on identifying the limitations of different demographics and developing tailored physical fitness plans while measuring the prevalence of CVD risk factors can be applied to the civilian population.

## Data Availability

The data that support the findings of this study are available publicly, published by the National Security Research Division in the 2018 Health Related Behavior Survey.
